# Exceptional response to chemo-immunotherapy in a patient with HER2-negative, TMB-high metastatic gastric mucinous adenocarcinoma: a case report and literature review

**DOI:** 10.3389/fimmu.2025.1713214

**Published:** 2026-01-14

**Authors:** Caiqi Liu, Xiangxue Li, Qi Qi, Fanjing Jing, Jing Lv, Wensheng Qiu, Shasha Wang

**Affiliations:** The Affiliated Hospital of Qingdao University, Qingdao, China

**Keywords:** conversion therapy, gastric cancer, HER2-negative, immunotherapy, TMB-H

## Abstract

Gastric mucinous adenocarcinoma (GMC) is a rare subtype of gastric cancer characterized by excessive mucus production, aggressive biological behavior, and poor prognosis, with most patients presenting with metastatic disease at initial diagnosis and losing the opportunity for curative resection. Currently, there are no standardized diagnostic and treatment guidelines for metastatic GMC in the conversion therapy setting, and the therapeutic effect of conventional chemotherapy remains unsatisfactory. Herein, we present a 69-year-old male patient diagnosed with HER2-negative, TMB-H advanced GMC, with intraperitoneal and retroperitoneal lymph node metastases. The patient was initially deemed unresectable by the multidisciplinary team (MDT) but opted for conversion therapy due to a strong willingness for treatment and good performance status (ECOG-PS=0). He received 6 cycles of FLOT chemotherapy combined with nivolumab, achieving partial response (PR) per RECIST 1.1. Subsequent laparoscopic distal gastric subtotal resection (D2+ lymphadenectomy) was performed, and postoperative pathology revealed a near pathological complete response (Mandard-TRG1) with no lymph node metastases (0/21), pathologically staged as ypTisN0. Postoperatively, the patient received 4 cycles of XELOX chemotherapy plus nivolumab, followed by consolidative radiotherapy synchronized with capecitabine and nivolumab, and subsequent maintenance therapy with capecitabine and nivolumab until sustained no evidence of disease (NED) was confirmed in January 2023. Regular surveillance, including the latest contrast-enhanced CT in May 2025, showed no recurrence or metastasis, with progression-free survival (PFS) exceeding 5 years. This exceptional and sustained response may be attributed to the synergistic effect of TMB-H and POLD1 mutation, which enhance neoantigen generation and sensitize tumors to immunotherapy. This case highlights the potential of biomarker-driven chemo-immunotherapy combined with MDT-guided multimodal treatment (surgery + adjuvant therapy + consolidative radiotherapy) to achieve curative intent in patients with metastatic GMC, providing valuable insights for personalized treatment strategies in this poor-prognosis population.

## 1 Introduction

Gastric cancer (GC) ranks as the fifth most commonly diagnosed malignancy and is the third leading cause of cancer-related mortality worldwide, posing a serious threat to human health (1). Mucinous adenocarcinoma, a subtype of malignant tumors originating from epithelial tissue, is distinguished by excessive mucus production. Histologically, it presents as substantial mucus accumulation either within or outside cancer cells. This type of carcinoma predominantly occurs in the stomach and intestines (2). In the past ten years, the considerable benefits associated with targeted therapy are primarily restricted to HER2-positive patients who receive trastuzumab in conjunction with chemotherapy (3). For HER2-negative metastatic GC patients, systemic chemotherapy has historically been regarded as first-line treatment. However, this approach has resulted in median overall survival (OS) rates of less than one year (4). With advancements in immune checkpoint inhibitors (ICIs), immunotherapy has yielded promising results and significantly improved outcomes for GC patients. A series of pivotal clinical trials have advanced the landscape of GC immunotherapy across different disease stages. CheckMate-649, a global Phase III study, demonstrated that nivolumab combined with chemotherapy significantly improved long-term survival in first-line advanced gastric/gastroesophageal junction (GEJ) cancer, with the Chinese subgroup achieving a 4-year overall survival (OS) rate of 25% in PD-L1 CPS≥5 patients and 21% in the overall population, far exceeding the chemotherapy-only group (5). ORIENT-16 confirmed that sintilimab plus chemotherapy prolonged median OS to 19.2 months (vs. 12.9 months) in PD-L1 CPS≥5 advanced gastric/GEJ adenocarcinoma patients and 15.2 months (vs. 12.3 months) in all randomized populations, with the 3-year OS rate doubling that of chemotherapy and a manageable safety profile (6). RATIONALE-305, a global Phase III trial, showed tislelizumab combined with chemotherapy improved median OS to 15.0 months (vs. 12.9 months) in the intent-to-treat population of HER2-negative advanced gastric/GEJ cancer and 16.4 months (vs. 12.8 months) in PD-L1 TAP≥5% patients, reducing the risk of death by 20%–29% (7). For resectable disease, MATTERHORN proved that perioperative durvalumab plus FLOT chemotherapy reduced the risk of disease progression, recurrence or death by 29%, with a 2-year event-free survival (EFS) rate of 67.4% (vs. 58.5% for chemotherapy alone) and a favorable OS trend (8); the DANTE trial found that adding atezolizumab to perioperative FLOT enhanced pathological downstaging and pathological complete response (pCR) rate (24% vs. 15%), especially in MSI or PD-L1 CPS≥10 subgroups, with comparable safety to chemotherapy (9); NEOSUMMIT-01, a Phase II trial, revealed that toripalimab combined with SOX/XELOX chemotherapy for locally advanced GC achieved a significantly higher major pathological response rate (44.4% vs. 20.4%) and pCR rate (22.2% vs. 7.4%), with striking benefits in dMMR patients and no increased perioperative safety risks (10). Nevertheless, a substantial proportion of GC patients exhibit insensitivity to ICIs treatments, presenting ongoing challenges for immunotherapy in cases of advanced GC. In this article, we present a case of metastatic GC with HER2-negative, TMB-H (Tumor Mutational Burden High) and POLD1 mutation that achieved a progression-free survival (PFS) of over 5 years after conversion therapy combining chemotherapy and immunotherapy (nivolumab). We also review the relevant literature the potential predictive roles of TMB-H and POLD1 mutations in GC immunotherapy response.

## 2 Case report

A 69-year-old male patient was admitted to our hospital on December 7, 2020, due to discomfort resulting from upper abdominal distension persisting for over seven months, with worsening symptoms over the past two weeks.

**Figure 1:**
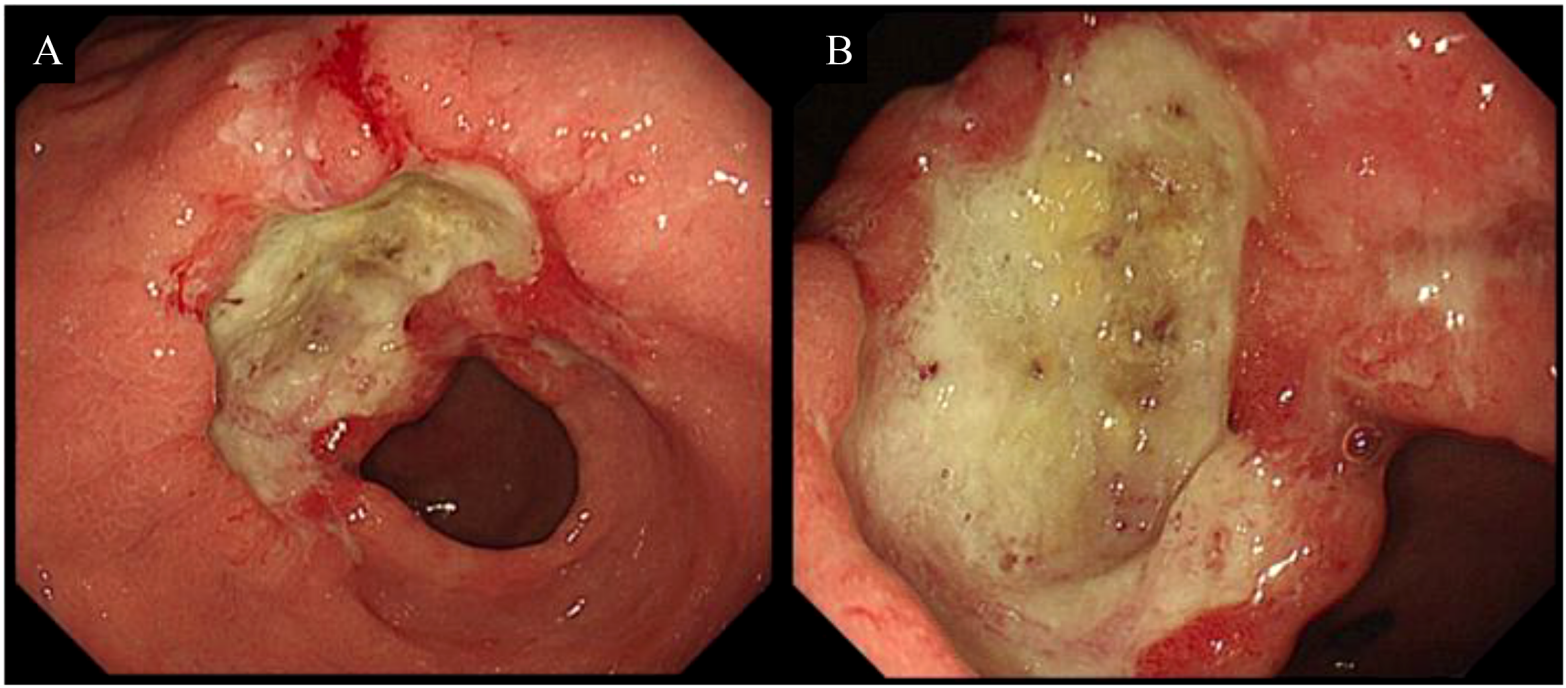
Baseline gastroscopy. **(A, B)** showing a large irregular mucosal defect at the gastric horn and lesser curvature of the antrum at baseline.

**Figure 2:**
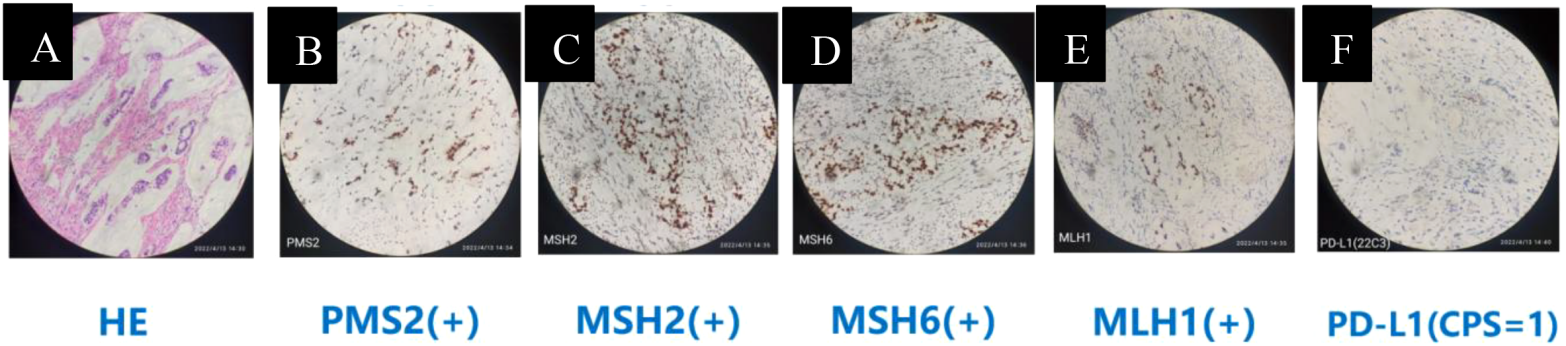
Immunohistochemistry at baseline. **(A)** HE stain. **(B-E)** PMS2, MSH2, MSH6, MLH1 stain (representing pMMR, nuclear positivity). **(F)** PD-L1 stain (CPS = 1, membranous staining in tumor cells and/or immune cells).

His medical history encompassing past medical, family, psychological, social history, and associated complications was unremarkable. Upon admission, physical examination disclosed no abnormalities. Laboratory tests indicated no signs of anemia or infection. Nevertheless, elevated levels of the tumor biomarker carbohydrate antigen 72-4 (CA72-4) were detected at 77.16 U/mL (normal value < 7 U/mL). Gastroscopy (November 26, 2020, **Figure 1**) unveiled a large, deep, and irregular ulcer at the gastric horn and lesser curvature of the antrum, with distinct boundaries. The surrounding mucosa exhibited irregular nodular protuberances with obvious surface ulceration and necrosis, covered with thick moss. The biopsy tissue was brittle and prone to bleeding, with a narrowed gastric antrum lumen and poor peristalsis in the gastric horn and antrum. Pathological analysis diagnosed poorly differentiated mucinous adenocarcinoma of the gastric antrum, categorized as Lauren diffuse type. Subsequently, immunohistochemistry (**Figure 2**) revealed proficient mismatch repair (pMMR) and PD-L1 positive (Combined Positive Score, CPS = 1). Molecular testing confirmed EBER and HER2 were negative.

**Figure 3:**
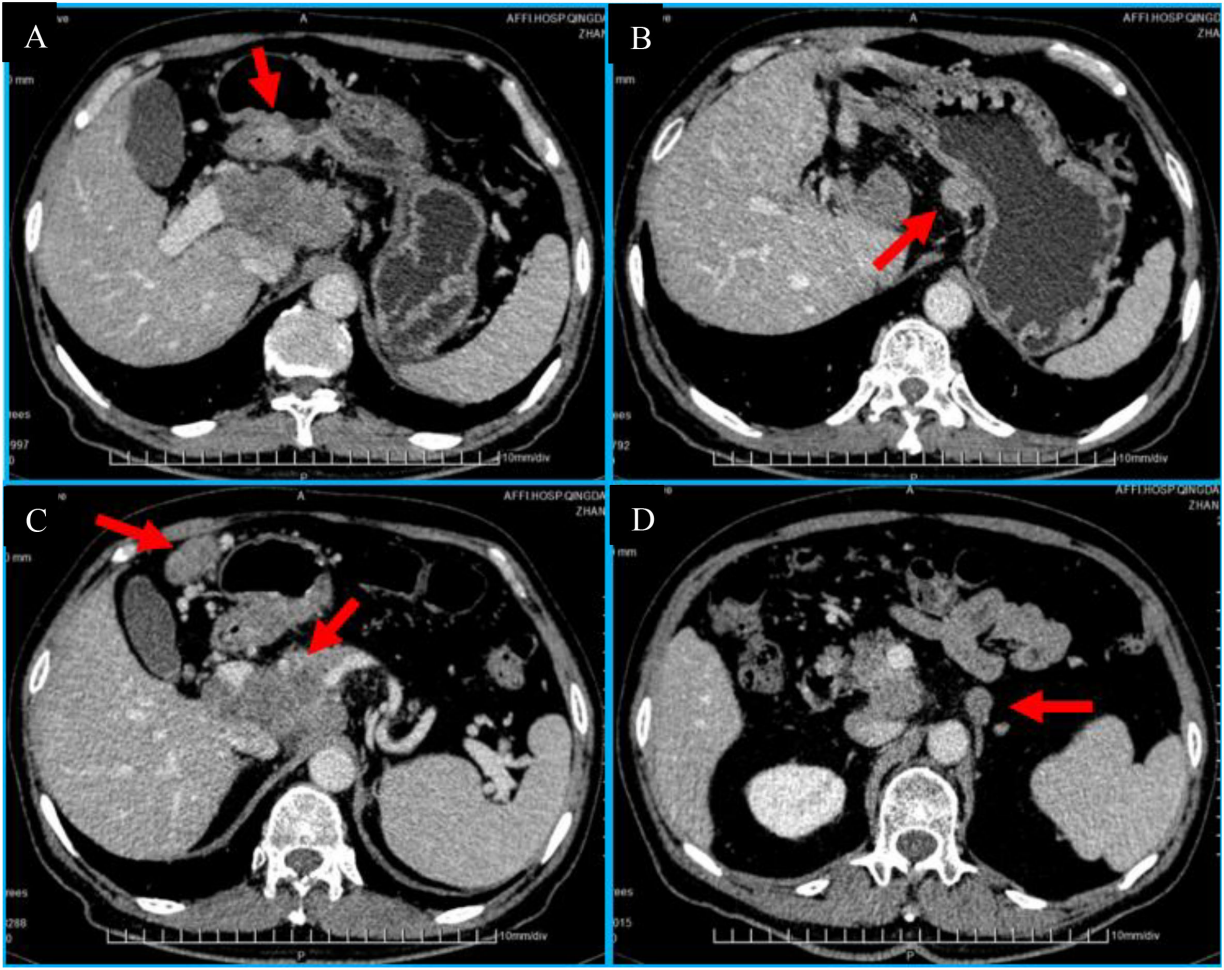
Baseline contrast-enhanced abdominal CT. **(A)** Axial image showing marked circumferential thickening of the gastric antrum wall (arrowhead). **(B)** Axial image demonstrating confluent bulky lymphadenopathy (arrows) encasing the portal vein (PV) and involving stations 7, 8, 9, 12, 13. **(C)** Coronal reconstruction image highlighting the extent of lymphadenopathy (arrows). **(D)** Group 16 lymph node metastasis.

An abdominal CT dynamic enhancement (**Figure 3**) demonstrated thickening of the antrum wall, penetrating the subserous connective tissue, and multiple enlarged lymph nodes including groups 3–9 and 12-14. Among them, the swollen lymph nodes in groups 7, 8, 9, 12, and 13 formed bulky II nodes and surrounded the portal vein. Metastasis was considered. To better guide the treatment, we requested the patient to undertake NGS detection and discovered TMB-H: 85.7 mutations/Mb, top 3.1%, POLD1 p.R1009H 24^th^ exon missense mutation.

The patient was diagnosed with metastatic gastric mucinous adenocarcinoma (cT3N3aM1, Stage IVB according to AJCC 8th edition), with metastases to both intraperitoneal and retroperitoneal lymph nodes. The multidisciplinary team (MDT) deemed the disease unresectable at presentation due to extensive lymph node involvement, particularly the encasement of Group 16 lymph node. According to patient’s strong willingness for treatment and currently good physical condition (ECOG-PS=0), conversion therapy with combined chemotherapy and immunotherapy was recommended, with the goal of downstaging the disease to enable potentially curative resection and achieve no evidence of disease (NED).

**Figure 4:**
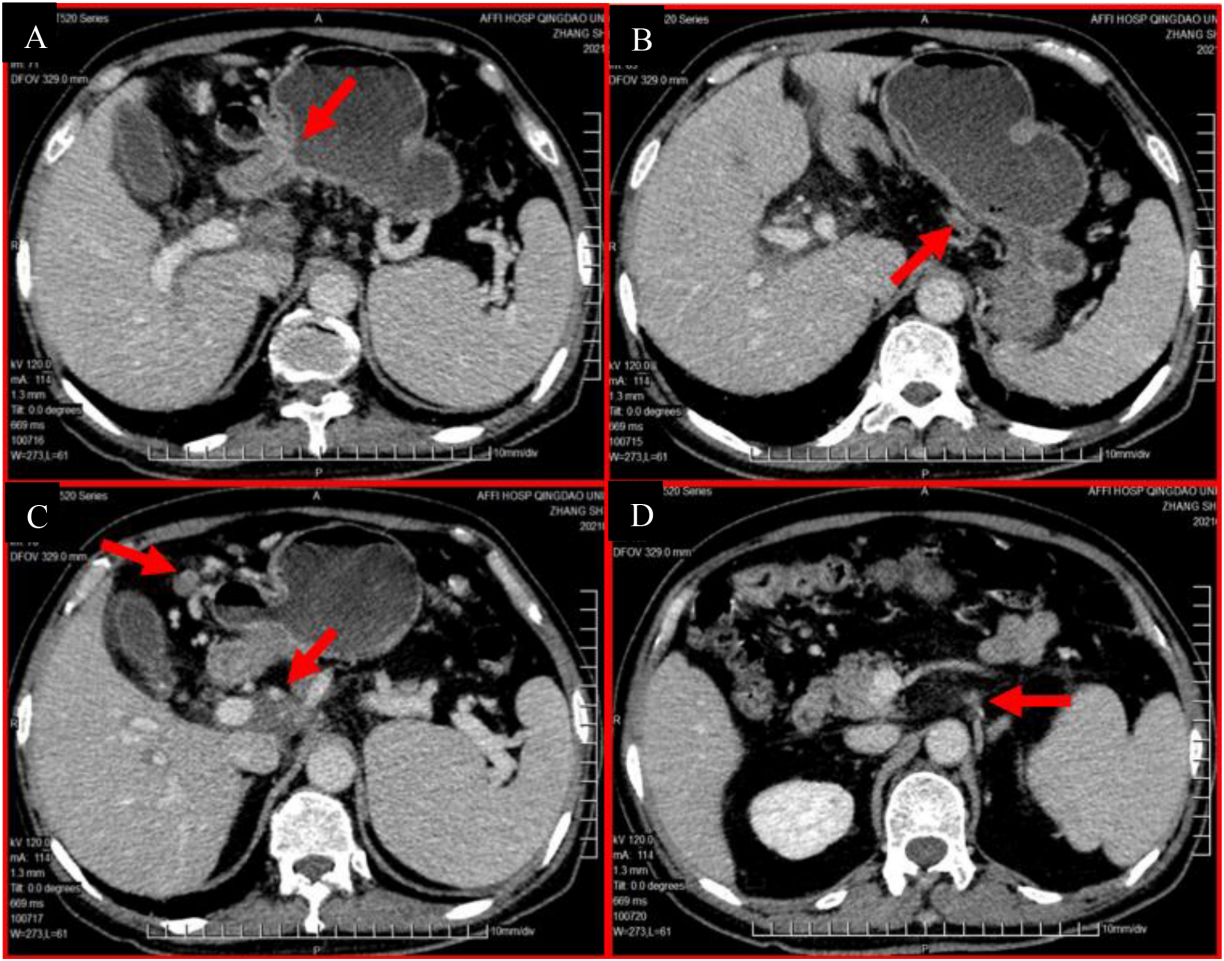
Restaging contrast-enhanced abdominal CT after completion of 6 cycles of conversion therapy (C6). **(A-D)**.

The patient underwent 6 cycles of conversion FLOT chemotherapy in combination with immunotherapy (albumin paclitaxel 170mg, oxaliplatin 150mg, calcium levofolinate 180mg, fluorouracil 4.75g civ46h, every 2 weeks and nivolumab 300mg every 3 weeks) from December 16, 2020 to March 11, 2021. The patient was well-tolerated and only occurred Grade I nausea and Grade II granulocytopenia. Meanwhile, the patient upper abdominal distension relieved and did not lose weight. A restaging CT scan and MDT review after 4 cycles (mid-treatment) demonstrated a remarkable partial response (PR, RECIST 1.1), with significant reduction in both the primary gastric tumor and metastatic lymphadenopathy. However, residual bulky lymphadenopathy persisted in the hepatic hilum/porta hepatis region (stations 12/13), with ill-defined planes adjacent to the portal vasculature, precluding safe surgical resection at that time. The MDT recommended completing the planned 6 cycles of chemo-immunotherapy. Restaging CT after cycle 6 (**Figure 4**) showed stable disease compared to the scan after cycle 4, with no further significant regression. Given the excellent initial response and stabilization, along with persistent but reduced/residual disease deemed potentially resectable, the MDT and patient opted for surgical intervention followed by adjuvant radiotherapy to the retroperitoneal nodal basin to consolidate the response and achieve NED.

The patient underwent laparoscopic palliative distal gastric subtotal resection (D2+, BII+Braun anastomosis) on April 1, 2021. Intraoperative laparoscopic exploration: no ascites, liver appeared normal without surface nodules, no visible peritoneal deposits. However, the tumor was located at the gastric angle and the lesser curvature of the antrum, with involvement of the serosa. The greater omentum was lifted to be closely apposed to the transverse colon, and the gastrocolic ligament was dissected using an ultrasonic scalpel, with the dissection extended leftward until reaching the splenic flexure of the colon; the left gastroepiploic vessels were dissected and mobilized on the pancreatic surface, and following transection of these vessels, lymph node dissection of station 4sb was performed before dissection was continued upward to mobilize the greater curvature of the stomach. The gastrocolic ligament was further dissected rightward to the hepatic flexure of the colon, the greater omentum was retracted toward the upper abdomen to expose the inferior margin of the pancreas, and upon identification of the fusion space, dissection was continued rightward to expose the pancreatic head, after which the right gastroepiploic artery and vein were dissected and transected, followed by lymph node dissection of station 6. The duodenal bulb was transected using an endoscopic linear cutter stapler at a distance of 2 cm distal to the pylorus; the common hepatic artery was dissected and exposed, and it was observed that Group 8 and Group 12 enlarged lymph nodes had encircled the portal vein and developed infiltration and fixation to the hepatic hilum, rendering them surgically unresectable. Silver clips were placed at the lesion sites for accurate localization in subsequent radiotherapy. Subsequently, the right gastric vessels were ligated and transected at the origin of the right gastric artery, the stomach was retracted upward to expose the superior margin of the pancreas, and the left gastric artery and splenic artery were dissected and mobilized, followed by lymph node dissection of stations 7, 9, and 11p; the coronary vein and left gastric vessels were then ligated and transected, the entire stomach was retracted en bloc toward the left upper quadrant of the abdomen, the hepatogastric ligament was ligated and transected to mobilize the lesser curvature of the stomach, and dissection was further extended upward to the right side of the cardia, where lymph node dissection of stations 1 and 3 was completed.

**Figure 5:**
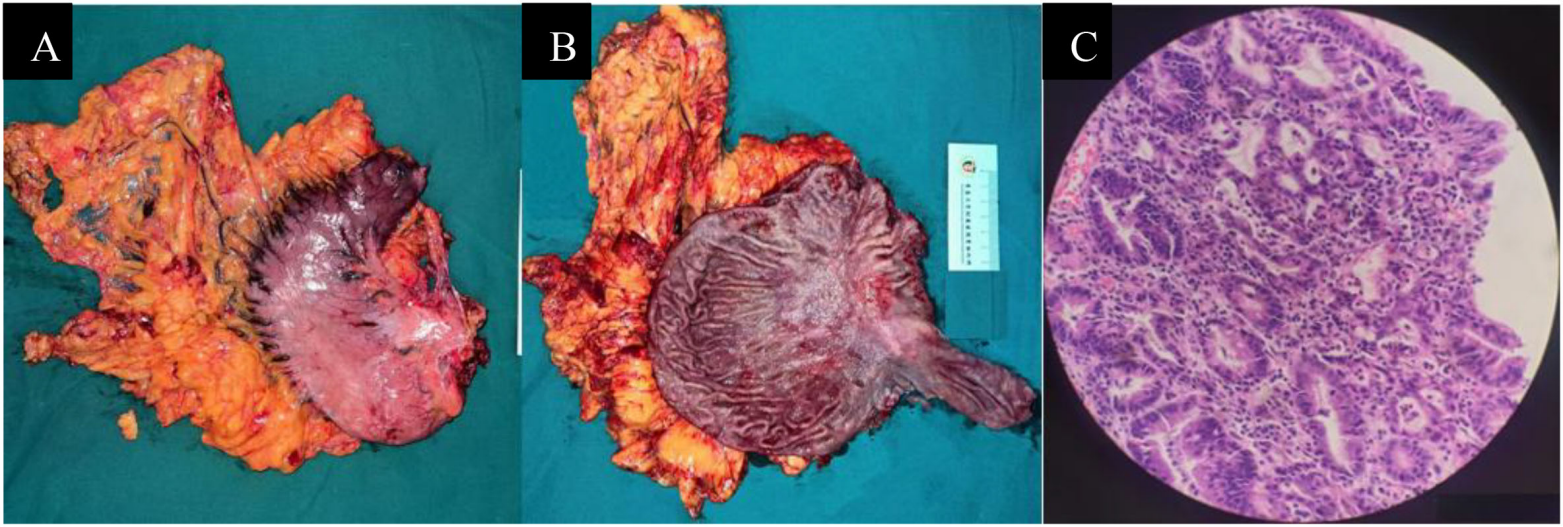
Surgical resection specimen and histopathology. **(A, B)** Gross photograph of the opened gastrectomy specimen showing the ulcer bed. **(C)** Photomicrograph (H&E stain, low power) demonstrating the ulcer bed with surrounding fibrosis and chronic inflammation.

The operation was successful. Postoperative pathology revealed moderate chronic inflammation with ulcer formation (measuring 2.5×2cm) and high-grade intraepithelial neoplasia in focal glands. The morphology of intramucosal carcinoma showed eosinophilic degeneration of cells, surrounded by hyperplastic interstitial fibrous tissue containing abundant lymphocytes, plasma cells, and some neutrophil infiltration, which was consistent with the changes observed after chemotherapy. The Tumor Regression Grade (Mandard-TRG) was Grade 1 (near-complete regression, residual tumor < 10% of the lesion area). No lymph node metastases were detected on either the lesser curvature side (0/13) or the greater curvature side (0/8). Pathological stage: ypTis (*in-situ* carcinoma within mucosa, reflecting residual treated disease) ypN0 (0/21). (AJCC 8th ed) (**Figure 5**).

**Figure 6:**
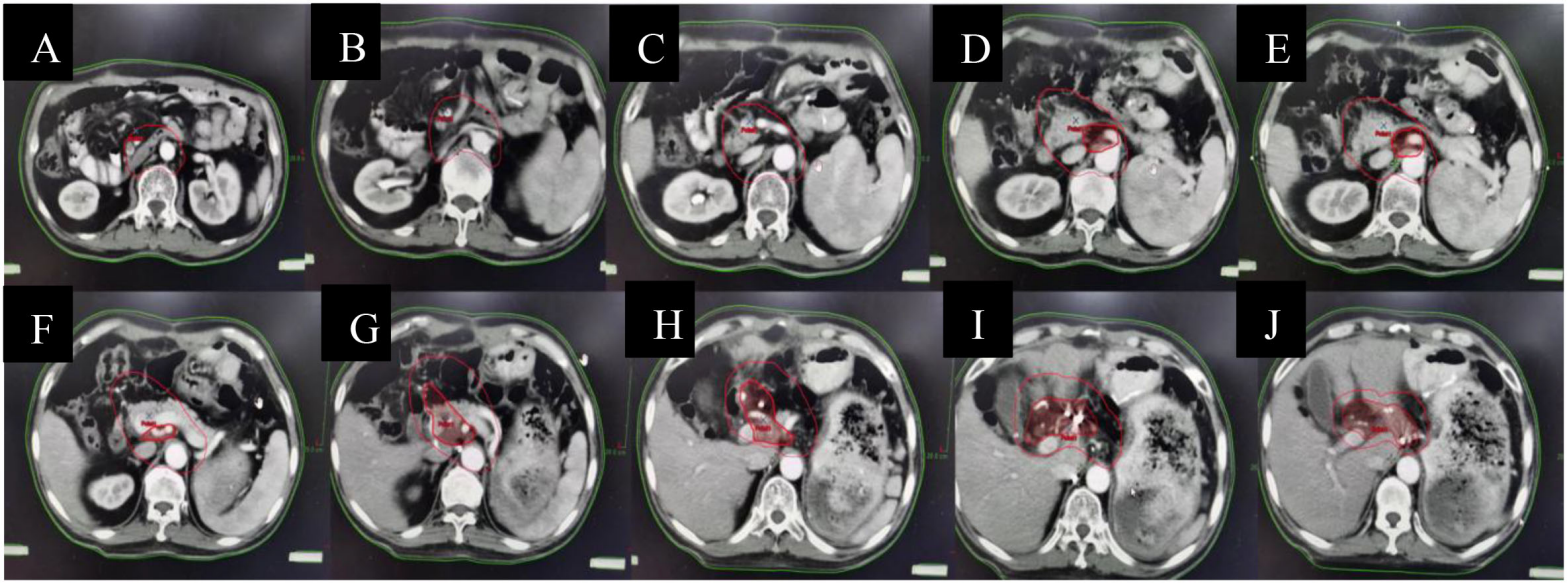
Radiotherapy planning. **(A-J)** The target areas included the hepatic portal, around the coeliac trunk and retroperitoneal lymph nodes. The target dose was PGTV 54Gy/25f/5f and PTV 45Gy/25f/5f.

Postoperatively, the patient experienced delayed gastric emptying (residual stomach retention) and fatigue. Following departmental discussion, we adjusted the therapeutic regimen to 4 cycles of XELOX chemotherapy (oxaliplatin 125mg on day 1 + capecitabine 1.5g twice daily from day 1-10, every 2 weeks) in combination with nivolumab immunotherapy (300mg iv every 3 weeks). Restaging CT on July 15th showed no evidence of recurrence or metastasis, consistent with ongoing response (PR). Subsequently, consolidative radiotherapy was administered to the high-risk nodal regions (hepatic hilum, periceliac axis, and retroperitoneum) (**Figure 6**) from August 4 to September 7, 2021, synchronized with capecitabine (825mg/m² twice daily on radiotherapy days) and nivolumab. The prescribed doses were: PGTV (involved nodes) 54 Gy in 25 fractions, PTV (elective nodal regions) 45 Gy in 25 fractions (2.16 Gy and 1.8 Gy per fraction respectively, 5 fractions per week). The treatment was well-tolerated with only Grade I gastrointestinal toxicity.

**Figure 7:**
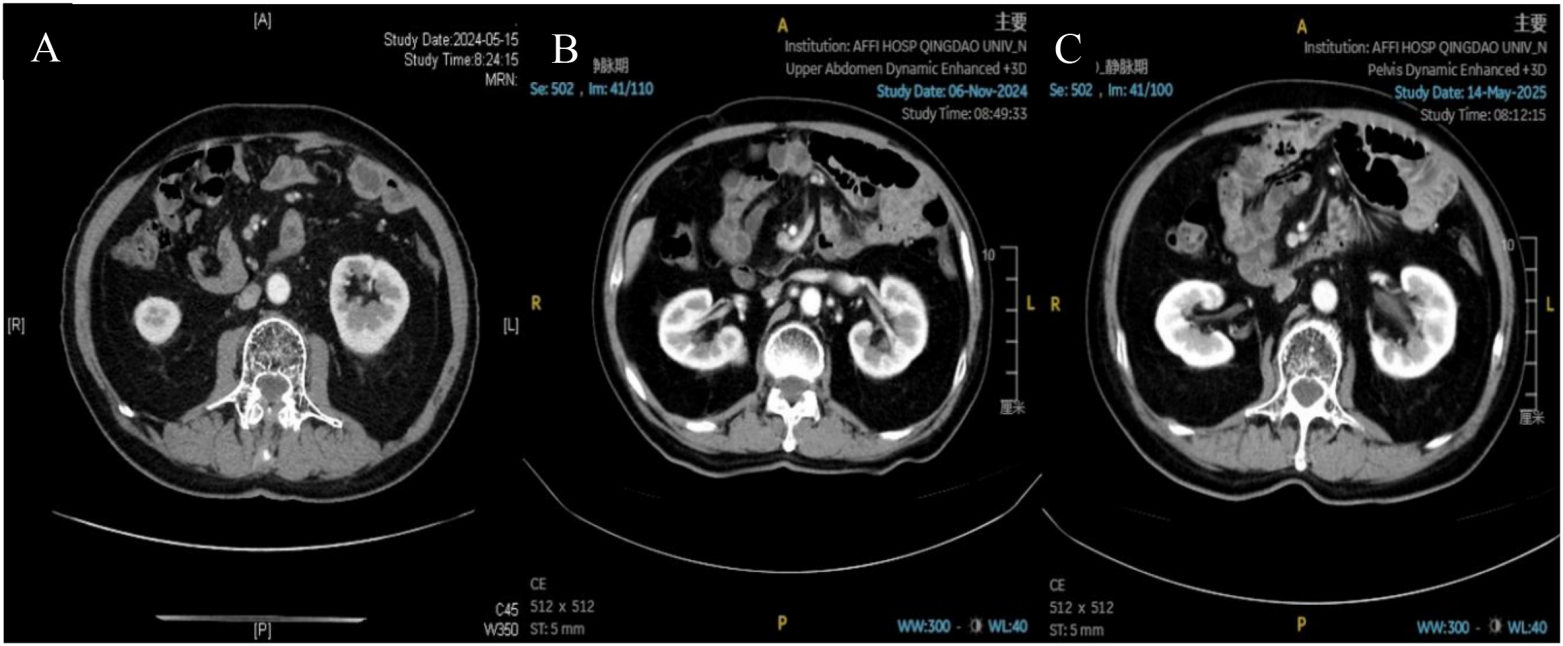
Surveillance abdominal CT scans. Images from May 2024 **(A)**, November 2024 **(B)**, and May 2025 **(C)** show stable post-treatment changes and no evidence of recurrent disease.

**Figure 8:**
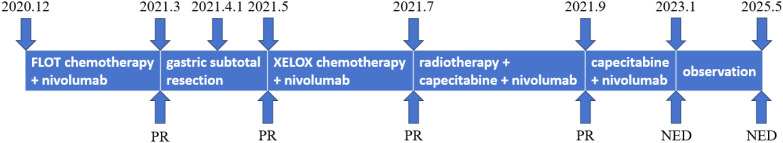
Treatment timeline. Schematic overview of therapeutic interventions, evaluations, and outcomes from diagnosis (Dec 2020) to last follow-up (May 2025). Key phases: Conversion Chemo-immunotherapy (FLOT + Nivo), Surgery, Adjuvant Therapy (XELOX + Nivo + RT), Maintenance Therapy (Cape + Nivo), and ongoing Surveillance. PFS >5 years and ongoing.

The patient continued maintenance therapy with capecitabine and nivolumab. Serial surveillance CT scans (chest and abdomen) confirmed sustained remission. A CT scan in January 2023 confirmed no evidence of disease (NED), prompting cessation of maintenance therapy. Subsequent regular examinations, including the most recent CT scans in May and November 2024, and May 2025 (**Figure 7**), continue to show no evidence of recurrence. The patient remains in good health with ongoing progression-free survival exceeding 5 years. The treatment timeline is summarized in **Figure 8**.

## 3 Discussion

The exceptional and sustained response observed in this case of initially unresectable metastatic GC can be attributed to the convergence of multiple factors: robust biomarker-driven patient selection, effective multimodal therapy, and a structured multidisciplinary team (MDT) approach.

### 3.1 Biomarker-driven patient selection: the role of TMB and POLD1

The recommendation of combined chemotherapy and immunotherapy was primarily supported by the results of the CheckMate-649 study presented at the European Society for Medical Oncology (ESMO) 2020 Congress, which demonstrated significant efficacy of immunotherapy combined with chemotherapy in improving the objective response rate and survival outcomes of patients with advanced gastric or gastroesophageal junction adenocarcinoma (5). Although the recommendation of this combined regimen was not based on TMB-H status, the patient’s ultra-high tumor mutational burden (TMB = 85.7 mut/Mb) and the presence of a POLD1 p.R1009H mutation are central to understanding the profound response to immunotherapy. TMB serves as a surrogate for neoantigen load, enabling enhanced immune recognition and T-cell activation (11). POLD1 encodes the catalytic subunit of DNA polymerase δ, a key enzyme involved in DNA replication and repair (12). The known oncogenic POLD1 mutations include E245K, E318K, D402N, L474P, S478N, L606M, L632M, R689W, and R1016H (13–20). Although the POLD1 p.R1009H variant is currently classified as a variant of uncertain significance (VUS), its location within the exonuclease domain—critical for DNA proofreading—strongly suggests functional significance (21). Defects in this domain are known to compromise replication fidelity, accelerate mutagenesis, and promote hypermutated phenotypes (22). A phase II clinical trial of toripalimab demonstrates that anti-PD-1 therapy elicited a favorable objective response (66.7%) in POLE/POLD1 exonuclease domain mutant tumors (23). The co-occurrence of this mutation with extreme TMB supports a synergistic mechanism wherein defective DNA repair amplifies neoantigen generation, thereby priming the tumor microenvironment for enhanced immune checkpoint inhibitors (ICIs) responsiveness (24–26).

Recent single-cell RNA sequencing (scRNA-seq) studies have provided unprecedented insights into the tumor immune landscape dynamics during immunotherapy. Huang et al. utilized scRNA-seq to map the immune cell composition and functional states in cancer patients receiving ICIs, revealing that TMB-H tumors are characterized by a distinct immune microenvironment enriched with clonally expanded effector CD8+ T cells, activated dendritic cells, and reduced myeloid-derived suppressor cells (MDSCs) (27). In this case, despite a relatively low PD-L1 CPS = 1, the ultra-high TMB (85.7 mut/Mb) likely reshaped the immune landscape into an “inflamed” phenotype—consistent with their findings that genetic hypermutability correlates with enhanced T-cell infiltration and functional activation. This scRNA-seq-derived evidence further validates our hypothesis that the concurrent TMB-H and POLD1 mutation confers exceptional ICI sensitivity, even in PD-L1-low metastatic GC.

The fundamental immunological insights from neoadjuvant ICI therapy, as elaborated by Pauken et al., further support the efficacy of our conversion therapy strategy (28). Their work demonstrated that neoadjuvant immunotherapy induced systemic T-cell priming and clonal expansion, with neoantigen-specific T cells persisting long-term to prevent recurrence—an effect particularly pronounced in tumors with high neoantigen loads (e.g., TMB-H tumors). In this case, the conversion chemo-immunotherapy not only achieved remarkable partial response but also elicited a durable immune memory response, as evidenced by >5 years of PFS. This aligns with Pauken et al.’s conclusion that neoadjuvant/conversion ICI-based regimens can unlock the full potential of anti-tumor immunity by targeting pre-existing neoantigens, which is especially relevant for hypermutated tumors.

Direct evidence supporting the prognostic and predictive value of POLD1 mutations in GC immunotherapy came from a prospective study by He et al., which analyzed circulating tumor DNA (ctDNA) in HER2-negative advanced GC patients receiving ICIs (29). Their findings demonstrated that co-occurring mutations involving POLD1 (e.g., IRS2/POLD1 or POLD1/CEBPA) were significantly associated with prolonged median PFS compared to patients without such co-mutations (p=0.006 and p=0.0315, respectively). Notably, this study highlighted that POLD1 mutations independently enhance ICI responsiveness by exacerbating genomic instability and increasing neoantigen presentation.

Further supporting this premise, recent pan-cancer analyses have demonstrated that patients with POLE/POLD1 mutations exhibit significantly prolonged survival following ICIs treatment compared to wild-type counterparts (30, 31). In gastrointestinal cancers specifically, these mutations are associated with a median overall survival of 34 months versus 18 months in non-mutated cases (32). This compelling clinical evidence reinforces the biological plausibility of POLD1-driven hypermutation as a predictive biomarker for immunotherapy efficacy, even when the specific variant remains functionally uncharacterized.

### 3.2 Rationale for treatment intensification and modality selection

In the conversion setting for locally advanced GC, the selection of chemotherapy backbones directly impacts the synergy with immunotherapy and clinical outcomes. The FLOT4-AIO trial demonstrated that the FLOT regimen achieved a 31.3% pathological complete response (pCR) rate and 65.8% R0 resection rate in neoadjuvant settings (33); its subgroup analysis showed that combining FLOT with PD-1 inhibitors (nivolumab) further elevated the objective response rate (ORR) to 78.2% and conversion resection rate to 71.5%, but was associated with a higher incidence of grade 3–4 neutropenia (42.1%) and fatigue (18.3%) (34).

In contrast, domestic phase II trials (e.g., the CAP 02 trial) focused on SOX (S-1 + oxaliplatin) and XELOX (capecitabine + oxaliplatin) combined with sintilimab (PD-1 inhibitor) reported ORRs of 72.3% and 69.8%, respectively, with conversion resection rates of 68.1% and 65.4%. These regimens show favorable safety profiles: grade 3–4 gastrointestinal toxicity (diarrhea, nausea) was only 12.5% (SOX) and 10.8% (XELOX), and oral administration of S-1/capecitabine improves patient compliance, making them more suitable for elderly patients or those with poor bone marrow reserve (35, 36).

Biomarker stratification further refines regimen selection: KEYNOTE-859 subgroup analysis indicated that in PD-L1 CPS ≥10 patients, FLOT + pembrolizumab prolonged median overall survival (OS) to 18.7 months, superior to SOX + pembrolizumab (15.2 months); while in microsatellite instability-high (MSI-H) patients, SOX/XELOX + immunotherapy achieved non-inferior OS (17.9 vs. 18.3 months) with lower toxicity (37). Current NCCN (2024 V1) and CSCO (2024) guidelines recommend FLOT + immunotherapy as the preferred option for fit patients with high tumor burden, and SOX/XELOX + immunotherapy for patients with comorbidities or limited tolerance.

The conversion therapy strategy of FLOT + nivolumab is further validated by the latest clinical research. Guo et al. conducted the phase II FDZL-GC001 trial, which showed that camrelizumab (a PD-1 inhibitor) combined with the Nab-POF regimen (nab-paclitaxel + oxaliplatin + fluorouracil) achieved an R0 resection rate of 75.0% and a 3-year OS rate exceeding 62.8% in patients with initially unresectable locally advanced or oligometastatic GC (38). Notably, patients with retroperitoneal lymph node metastasis (accounting for 40.4% of the study population) also achieved favorable outcomes. This further confirms the sensitivity and effectiveness of chemotherapy + PD-1 inhibitor as the core regimen for conversion therapy in metastatic GC.

The surgical outcome and long-term survival of conversion surgery in advanced GC has also been reported. Chen etc. retrospectively reviewed 95 primary advanced gastric adenocarcinoma patients who underwent systemic chemotherapy and conversion surgery and discovered that advanced GC patients might obtain a survival benefit from conversion surgery. Performing a sufficient number of cycles of induction chemotherapy (usually ≥ 6 cycles) was recommended (39). Nobuhiro Nakazawa etc. also reported the conversion surgery transition rate after chemotherapy plus nivolumab as a first-line treatment for unresectable advanced or recurrent GC was 11.5%, correlating with a good ECOG-PS (40).

These findings suggest that 6 cycles of FLOT regimen + immunotherapy may be a promising option for the conversion treatment of metastatic gastric adenocarcinoma. Given that the patient has a good performance status, no underlying diseases, and a strong willingness to undergo surgery, we opted for the three-drug FLOT chemotherapy combined with nivolumab to increase the R0 resection rate.

Following conversion surgery, the patient achieved a near pathological complete response (TRG1) with node-negative status (0/21). However, Group 8 and Group 12 enlarged lymph nodes were surgically unresectable. The subsequent administration of adjuvant radiotherapy (54 Gy) aimed to eradicate potential metastases in unresectable and high-risk regions. Radiotherapy synergizes with surgery and systemic therapy through multiple mechanisms: chemotherapy induces G2/M cell cycle arrest, increasing radiotherapy sensitivity by 1.5-fold (41); surgery reduces primary tumor burden, narrowing radiotherapy target volume and decreasing normal tissue exposure (42); immunotherapy enhances radiotherapy-induced immunogenic cell death, promoting abscopal effect (43).

The synergistic immunomodulatory effect of radiotherapy combined with nivolumab in GC is further validated by Mimura et al.’s study, which demonstrated that oligo-fractionated irradiation induces multiple immune-sensitizing mechanisms: *de novo* tumor-associated antigen (TAA)-specific CD8+ T cell generation via antigen spreading, enrichment of CD45RO+CD27+CD127+ central memory T cells in non-progressors, preservation of T cell receptor β (TCRβ) repertoire diversity to overcome tumor heterogeneity, and significant reduction in circulating tumor DNA (ctDNA)-derived TMB, reflecting effective tumor clearance (44). Notably, the study confirmed the safety of this combination (no additional grade ≥3 toxicity), consistent with our patient’s favorable tolerance.

The 54 Gy dose for retroperitoneal residual nodal disease was determined based on evidence-based dose-response relationships. The INT 0116 trial long-term follow-up (15-year) showed that 50–54 Gy consolidative radiotherapy reduced local recurrence rate by 42% compared to 45 Gy, with nodal sterilization rate increasing from 58% to 76% in patients with macroscopic residual nodes (45, 46). ASTRO/ESTRO gastric cancer radiotherapy guidelines (2023) explicitly recommend 54 Gy as the standard dose for residual nodal lesions, as doses exceeding 54 Gy do not further improve local control but increase grade ≥3 toxicity by 2.3-fold.

A 2025 meta-analysis (involving 18 studies and 8923 patients, with 32% focusing on residual nodal disease) confirmed that consolidative radiotherapy for post-systemic therapy residual lesions (including retroperitoneal nodes) significantly improved local recurrence-free survival (LRFS, HR = 0.63, 95%CI 0.55–0.72) and OS (HR = 0.77, 95%CI 0.70–0.85). Subgroup analysis specifically highlighted retroperitoneal nodal involvement as a high-benefit population: LRFS improvement reached 40% (vs. 31% for perigastric nodes). Meanwhile, potential toxicities were clinically acceptable: grade ≥3 toxicity occurred in 27.6% of patients and no grade ≥4 toxicity was reported (47). In this case, the patient was well-tolerated with only grade 1 gastrointestinal toxicity.

Nevertheless, the omission of pre-radiotherapy PET-CT represents a limitation, as metabolic imaging could have refined patient selection for consolidative irradiation by identifying residual metabolic activity that might guide radiotherapy fields and dose escalation (48).

### 3.3 Implications of ultra-high TMB and adaptive treatment strategies

This case raises important questions regarding TMB interpretation in GC. While a cutoff of ≥10 mut/Mb is widely adopted from KEYNOTE-158 (49), ultra-high TMB values (e.g., >80 mut/Mb) may represent a distinct biological entity—the hypermutator phenotype—with potentially enhanced responsiveness to immunotherapy. This suggests that TMB might be better utilized as a continuous variable rather than a dichotomous biomarker, with extremely high values potentially warranting more aggressive immunotherapy approaches or treatment de-escalation in sustained responders (50).

The integration of MDT-based decision-making throughout the treatment course was indispensable to achieving coordinated and curative-intent therapy (51, 52). Through three dedicated MDT meetings, consensus was reached on critical junctures including the timing of surgical intervention, the need for radiotherapy, and the duration of systemic therapy. This collaborative approach exemplifies how complex cases benefit from integrated expertise across medical oncology, radiation oncology, surgery, and diagnostic disciplines.

### 3.4 The decision to stop treatment after NED

The duration of maintenance immunotherapy in exceptional responders remains poorly defined (53). Our decision to discontinue treatment after sustained NED was based on a multidisciplinary and individualized risk assessment framework, which incorporates the following key factors.

Definitive control of local and regional disease: The patient underwent radical gastrectomy and adjuvant radiotherapy targeting the unresectable and high-risk area (retroperitoneal lymph nodes). Definitive local intervention was achieved, laying a solid foundation for the subsequent suspension of systemic therapy.

Adequacy of systemic therapy and evidence of sustained remission: The patient completed 2 years of standardized immunotherapy followed by maintenance therapy. During the multi-year treatment and follow-up period, multiple re-evaluations (including tumor markers and contrast-enhanced CT) consistently revealed no evidence of tumor recurrence or metastasis, which provides key support for confirming the “disease-free state”.

Pre-discontinuation re-evaluation protocol and the patient’s individual circumstances: We originally planned to perform a more precise re-evaluation via PET-CT prior to treatment discontinuation, but this was temporarily delayed due to the patient’s personal and family circumstances. Given the patient’s long-term and stable clinical and imaging remission, MDT conducted a thorough discussion and concluded that continuing the suspension of systemic therapy with close follow-up presents a favorable benefit-risk profile.

### 3.5 Limitations and future directions

As a single-case report, our findings necessitate validation in larger cohorts. The predictive utility of TMB across GC subtypes requires further standardization, and functional studies are needed to clarify the role of POLD1 p.R1009H and similar VUS in driving hypermutation. Additionally, while we observed a remarkable response, primary and acquired resistance to immunotherapy remains a significant challenge for most metastatic GC patients (54), emphasizing the need for better understanding of resistance mechanisms within the tumor microenvironment.

Moving forward, we propose a structured pathway for oligometastatic GC conversion therapy: 1. MDT Evaluation: Comprehensive staging and biomarker profiling including NGS and PD-L1 testing; 2. Risk Stratification: Prioritize immunotherapy in TMB-H, MSI-H, or POLE/POLD1-mutant cases; 3. Conversion Therapy: Chemo-immunotherapy ± local modalities based on tumor location and burden; 4. Consolidative Treatment: Surgery and/or radiotherapy for responders achieving disease control; 5. Response-Adapted Maintenance: Tailored therapy based on metabolic and pathologic response.

Future efforts should focus on standardizing biomarker assays, validating functional mutations through laboratory models, and integrating dynamic monitoring tools such as ctDNA to guide therapy duration and detect recurrence earlier. Additionally, rational combinations—such as ICIs with PARP inhibitors in DNA repair-deficient GC—warrant clinical evaluation in selected populations.

## 4 Conclusion

This case exemplifies the transformative potential of precision immuno-oncology in metastatic GC, demonstrating how biomarker-driven therapy selection can lead to unprecedented outcomes even in traditionally poor-prognosis disease. It underscores the importance of integrating molecular profiling with multidisciplinary management to deliver personalized, curative-intent therapy. As we enter an era of increasingly sophisticated cancer management, such exceptional responses provide both hope and direction for optimizing treatment strategies in molecularly selected populations.

## Data Availability

The original contributions presented in the study are included in the article/supplementary material. Further inquiries can be directed to the corresponding authors.

## References

[1] Carmo GCD Cavalcante RM Aquino TMFD . Gastric cancer: an overview. Rev da Associação Médica Bras. (2024) 70:e2024S116. doi: 10.1590/1806-9282.2024s116, PMID: 38865536 PMC11164282

[2] Tseng CH Fang WL Huang KH Chen MH Chao Y Lo SS . The clinicopathological characteristics and genetic alterations of mucinous carcinoma of the stomach. J Chin Med Assoc. (2020) 83:141–147. doi: 10.1097/JCMA.0000000000000232, PMID: 32015267 PMC13047988

[3] Zhu Y Zhu X Wei X Tang C Zhang W . HER2-targeted therapies in gastric cancer. Biochim Biophys Acta (BBA) - Rev Cancer. (2021) 1876:188549. doi: 10.1016/j.bbcan.2021.188549, PMID: 33894300

[4] Yao Y Deng R Liao D Xie H Zuo J Jia Y . Maintenance treatment in advanced HER2-negative gastric cancer. Clin Trans Oncol. (2020) 22:2206–12. doi: 10.1007/s12094-020-02379-7, PMID: 32562198

[5] Janjigian YY Shitara K Moehler M Garrido M Salman P Shen L . First-line nivolumab plus chemotherapy versus chemotherapy alone for advanced gastric, gastro-oesophageal junction, and oesophageal adenocarcinoma (CheckMate 649): a randomised, open-label, phase 3 trial. Lancet (London England). (2021) 398:27–40. doi: 10.1016/S0140-6736(21)00797-2, PMID: 34102137 PMC8436782

[6] Xu J Jiang H Pan Y Gu K Cang S Han L . Sintilimab plus chemotherapy for unresectable gastric or gastroesophageal junction cancer: the ORIENT-16 randomized clinical trial. JAMA. (2023) 330:2064–74. doi: 10.1001/jama.2023.19918, PMID: 38051328 PMC10698618

[7] Qiu M Oh DY Kato K Arkenau T Tabernero J Correa MC . Tislelizumab plus chemotherapy versus placebo plus chemotherapy as first line treatment for advanced gastric or gastro-oesophageal junction adenocarcinoma: RATIONALE-305 randomised, double blind, phase 3 trial. BMJ (Clinical Res ed.). (2024) 385:e078876–e078876. doi: 10.1136/bmj-2023-078876, PMID: 38806195

[8] Janjigian YY Van Cutsem E Muro K Wainberg Z Al-Batran SE Hyung WJ . MATTERHORN: phase III study of durvalumab plus FLOT chemotherapy in resectable gastric/gastroesophageal junction cancer. Future Oncol (London England). (2022) 18:2465–73. doi: 10.2217/fon-2022-0093, PMID: 35535555

[9] Lorenzen S Götze TO Thuss-Patience P Biebl M Homann N Schenk M . Perioperative atezolizumab plus fluorouracil, leucovorin, oxaliplatin, and docetaxel for resectable esophagogastric cancer: interim results from the randomized, multicenter, phase II/III DANTE/IKF-s633 trial. J Clin Oncol Off J Am Soc Clin Oncol. (2024) 42:410–20. doi: 10.1200/JCO.23.00975, PMID: 37963317

[10] Yuan S Nie RC Jin Y Liang CC Li YF Jian R . Perioperative toripalimab and chemotherapy in locally advanced gastric or gastro-esophageal junction cancer: a randomized phase 2 trial. Nat Med. (2024) 30:552–9. doi: 10.1038/s41591-023-02721-w, PMID: 38167937

[11] Jardim DL Goodman A de Melo Gagliato D Kurzrock R . The challenges of tumor mutational burden as an immunotherapy biomarker. Cancer Cell. (2021) 39:154–73. doi: 10.1016/j.ccell.2020.10.001, PMID: 33125859 PMC7878292

[12] Gola M Stefaniak P Godlewski J Jereczek-Fossa BA Starzyńska A . Prospects of POLD1 in human cancers: A review. Cancers (Basel). (2023) 15:1905. doi: 10.3390/cancers15061905, PMID: 36980791 PMC10047664

[13] Palles C Cazier JB Howarth KM Domingo E Jones AM Broderick P . Germline mutations affecting the proofreading domains of POLE and POLD1 predispose to colorectal adenomas and carcinomas. Nat Genet. (2013) 45:136–44. doi: 10.1038/ng.2503, PMID: 23263490 PMC3785128

[14] Clausen AR Zhang S Burgers PM Lee MY Kunkel TA . Ribonucleotide incorporation, proofreading and bypass by human DNA polymerase delta. DNA Repair (Amst). (2013) 12:121–7. doi: 10.1016/j.dnarep.2012.11.006, PMID: 23245697 PMC3552135

[15] Shlien A Campbell BB de Borja R Alexandrov LB Merico D Wedge D . Combined hereditary and somatic mutations of replication error repair genes result in rapid onset of ultra-hypermutated cancers. Nat Genet. (2015) 47:257–62. doi: 10.1038/ng.3202, PMID: 25642631

[16] Giannakis M Mu XJ Shukla SA Qian ZR Cohen O Nishihara R . Genomic correlates of immune-cell infiltrates in colorectal carcinoma. Cell Rep. (2016) 15:857–65. doi: 10.1016/j.celrep.2016.03.075, PMID: 27149842 PMC4850357

[17] Campbell BB Light N Fabrizio D Zatzman M Fuligni F de Borja R . Comprehensive analysis of hypermutation in human cancer. Cell. (2017) 171:1042–1056.e10. doi: 10.1016/j.cell.2017.09.048, PMID: 29056344 PMC5849393

[18] Robinson PS Coorens THH Palles C Mitchell E Abascal F Olafsson S . Increased somatic mutation burdens in normal human cells due to defective DNA polymerases. Nat Genet. (2021) 53:1434–42. doi: 10.1038/s41588-021-00930-y, PMID: 34594041 PMC8492474

[19] Wei CH Wang EW Ma L Zhou Y Zheng L Hampel H . POLD1 DEDD motif mutation confers hypermutation in endometrial cancer and durable response to pembrolizumab. Cancers (Basel). (2023) 15:5674. doi: 10.3390/cancers15235674, PMID: 38067377 PMC10705788

[20] Andrianova MA Seplyarskiy VB Terradas M Sánchez-Heras AB Mur P Soto JL . Discovery of recessive effect of human polymerase delta proofreading deficiency through mutational analysis of POLD1-mutated normal and cancer cells. Eur J Hum Genet. (2024) 32:837–45. doi: 10.1038/s41431-024-01598-8, PMID: 38658779 PMC11219999

[21] Tsarin L Shcherbakova PV . PolED: a manually curated database of functional studies of POLE and POLD1 variants reported in humans. Database (Oxford). (2025) 2025:baaf076. doi: 10.1093/database/baaf076, PMID: 41263451 PMC12631556

[22] Zhu M Cui H Zhang L Zhao K Jia X Jin H . Assessment of POLE and POLD1 mutations as prognosis and immunotherapy biomarkers for stomach adenocarcinoma. Trans Cancer Res. (2022) 11:193–205. doi: 10.21037/tcr-21-1601, PMID: 35261896 PMC8841685

[23] Jin Y Huang RJ Guan WL Wang ZQ Mai ZJ Li YH . A phase II clinical trial of toripalimab in advanced solid tumors with polymerase epsilon/polymerase delta (POLE/POLD1) mutation. Signal transduction targeted Ther. (2024) 9:227–7. doi: 10.1038/s41392-024-01939-5, PMID: 39218995 PMC11366758

[24] Marabelle A Fakih M Lopez J Shah M Shapira-Frommer R Nakagawa K . Association of tumour mutational burden with outcomes in patients with advanced solid tumours treated with pembrolizumab: prospective biomarker analysis of the multicohort, open-label, phase 2 KEYNOTE-158 study. Lancet Oncol. (2020) 21:1353–65. doi: 10.1016/S1470-2045(20)30445-9, PMID: 32919526

[25] Wang F Wei XL Wang FH Xu N Shen L Dai GH . Safety, efficacy and tumor mutational burden as a biomarker of overall survival benefit in chemo-refractory gastric cancer treated with toripalimab, a PD-1 antibody in phase Ib/II clinical trial NCT02915432. Ann Oncol. (2019) 30:1479–86. doi: 10.1093/annonc/mdz197, PMID: 31236579 PMC6771223

[26] Shitara K Özgüroğlu M Bang YJ Di Bartolomeo M Mandalà M Ryu MH . The association of tissue tumor mutational burden (tTMB) using the Foundation Medicine genomic platform with efficacy of pembrolizumab versus paclitaxel in patients (pts) with gastric cancer (GC) from KEYNOTE-061. J Clin Oncol. (2020) 38:4537–7. doi: 10.1200/JCO.2020.38.15_suppl.4537

[27] Huang Y Zhang M Gao Q . Mapping the tumor immune landscape: single-cell RNA sequencing in cancer immunotherapy. Cancer Lett. (2025) 633:218012. doi: 10.1016/j.canlet.2025.218012, PMID: 40889736

[28] Pauken KE Alhalabi O Goswami S Sharma P . Neoadjuvant immune checkpoint therapy: Enabling insights into fundamental human immunology and clinical benefit. Cancer Cell. (2025) 43:623–40. doi: 10.1016/j.ccell.2025.03.005, PMID: 40118048 PMC13143226

[29] He M Ji C Li Z Chen S Gao J Shen L . Circulating tumor DNA predicts clinical benefits of immune checkpoint blockade in HER2-negative patients with advanced gastric cancer. Gastric cancer: Off J Int Gastric Cancer Assoc Japanese Gastric Cancer Assoc. (2025) 28:872–85. doi: 10.1007/s10120-025-01621-x, PMID: 40372586 PMC12378147

[30] Wang F Zhao Q Wang YN Jin Y He MM Liu ZX . Evaluation of POLE and POLD1 mutations as biomarkers for immunotherapy outcomes across multiple cancer types. JAMA Oncol. (2019) 5:1504–6. doi: 10.1001/jamaoncol.2019.2963, PMID: 31415061 PMC6696731

[31] Ying J Yang L Yin JC Xia G Xing M Chen X . Additive effects of variants of unknown significance in replication repair-associated DNA polymerase genes on mutational burden and prognosis across diverse cancers. J ImmunoTherapy Cancer. (2021) 9:e002336. doi: 10.1136/jitc-2021-002336, PMID: 34479923 PMC8420654

[32] Liu Y Hu P Xu L Zhang X Li Z Li Y . Current progress on predictive biomarkers for response to immune checkpoint inhibitors in gastric cancer: how to maximize the immunotherapeutic benefit? Cancers. (2023) 15:2273. doi: 10.3390/cancers15082273, PMID: 37190201 PMC10137150

[33] Al-Batran S Homann N Pauligk C Goetze TO Meiler J Kasper S . Perioperative chemotherapy with fluorouracil plus leucovorin, oxaliplatin, and docetaxel versus fluorouracil or capecitabine plus cisplatin and epirubicin for locally advanced, resectable gastric or gastro-oesophageal junction adenocarcinoma (FLOT4): a randomised, phase 2/3 trial. Lancet (London England). (2019) 393:1948–57. doi: 10.1016/S0140-6736(18)32557-1, PMID: 30982686

[34] Grimm M Grün CB Niegisch G Pichler M Roghmann F Schmitz-Dräger B . Tailored immunotherapy approach with nivolumab in advanced transitional cell carcinoma (TITAN-TCC). J Clin Oncol. (2022) 40:441–1. doi: 10.1200/JCO.2022.40.6_suppl.441 35275706

[35] Ding X Wang Xuejun Li Bin Wang Longgang Guo Honghai Shang Liang . PERSIST: A multicenter, randomized phase II trial of perioperative oxaliplatin and S-1 (SOX) with or without sintilimab in resectable locally advanced gastric/gastroesophageal junction cancer (GC/GEJC). J Clin Oncol. (2023) 41:364–4. doi: 10.1200/JCO.2023.41.4_suppl.364

[36] Guo H Ding P Sun C Yang P Tian Y Liu Y . Efficacy and safety of sintilimab plus XELOX as a neoadjuvant regimen in patients with locally advanced gastric cancer: A single-arm, open-label, phase II trial. Front Oncol. (2022) 12:927781–1. doi: 10.3389/fonc.2022.927781, PMID: 36091139 PMC9458882

[37] Doufas AG Tian L Kutscher S Finnsson E Ágústsson JS Chung BI . The effect of hyperoxia on ventilation during recovery from general anesthesia: A randomized pilot study for a parallel randomized controlled trial. J Clin Anesth. (2022) 83:110982–2. doi: 10.1016/j.jclinane.2022.110982, PMID: 36265267

[38] Feng W Huang H Zhao X Fu H Zhou Y Zhang W . Conversion effects of PD-1 inhibitor camrelizumab (Cam) combined with Nab-POF regimen in patients (pts) with initially unresectable locally advanced or limited metastatic gastric or gastroesophageal junction adenocarcinoma: FDZL-001 trial. J Clin Oncol. (2025) 43:334–4. doi: 10.1200/JCO.2025.43.4_suppl.334

[39] Chen G Yuan SQ Nie RC Luo TQ Jiang KM Liang CC . Surgical outcome and long-term survival of conversion surgery for advanced gastric cancer. Ann Surg Oncol. (2020) 27:4250–60. doi: 10.1245/s10434-020-08559-7, PMID: 32506192

[40] Nakazawa N Sohda M Hosoi N Watanabe T Kumakura Y Yamashita T . Conversion surgery after chemotherapy plus nivolumab as the first-line treatment for unresectable advanced or recurrent gastric cancer and a biomarker study using the gustave roussy immune score: A multicenter study. Ann Surg Oncol. (2024) 31:9023–9. doi: 10.1245/s10434-024-16161-4, PMID: 39225857

[41] Dillon MT Good JS Harrington KJ . Selective targeting of the G2/M cell cycle checkpoint to improve the therapeutic index of radiotherapy. Clin Oncol (Royal Coll Radiologists (Great Britain)). (2014) 26:257–65. doi: 10.1016/j.clon.2014.01.009, PMID: 24581946

[42] Chen Q Deng Y Wang K Li Y Bi X Li K . Dynamic prognostic models for colorectal cancer with liver metastases. JAMA Network Open. (2025) 8:e2529093. doi: 10.1001/jamanetworkopen.2025.29093, PMID: 40864468 PMC12391993

[43] Huang J Theelen WSME Belcaid Z Najjar M van der Geest D Singh D . Combination of pembrolizumab and radiotherapy induces systemic antitumor immune responses in immunologically cold non-small cell lung cancer. Nat Cancer. (2025) 6:1676–92. doi: 10.1038/s43018-025-01018-w, PMID: 40696153 PMC12559004

[44] Mimura K Ogata T Nguyen PHD Roy S Kared H Yuan YC . Combination of oligo-fractionated irradiation with nivolumab can induce immune modulation in gastric cancer. J ImmunoTherapy Cancer. (2024) 12:e008385. doi: 10.1136/jitc-2023-008385, PMID: 38290769 PMC10828861

[45] Macdonald JS Smalley SR Benedetti J Hundahl SA Estes NC Stemmermann GN . Chemoradiotherapy after surgery compared with surgery alone for adenocarcinoma of the stomach or gastroesophageal junction. New Engl J Med. (2001) 345:725–30. doi: 10.1056/NEJMoa010187, PMID: 11547741

[46] Kozak KR Moody JS . The survival impact of the intergroup 0116 trial on patients with gastric cancer. Int J Radiat oncology biology Phys. (2008) 72:517–21. doi: 10.1016/j.ijrobp.2007.12.029, PMID: 18249500

[47] Wang Z Dong L Shi W Gao L Jiang X Xue S . Postoperative therapy for local-advanced gastric cancer: A systematic review and meta-analysis. Adv Clin Exp medicine: Off Organ Wroclaw Med Univ. (2024) 33:669–78. doi: 10.17219/acem/171616, PMID: 38085005

[48] Li M Xuan G Gu H Wu J Wang Y . Survival benefit associated with postoperative PET–CT before adjuvant radiotherapy or chemoradiotherapy in patients with oral squamous cell carcinoma. Int J Oral Maxillofac Surg. (2022) 51:1382–8. doi: 10.1016/j.ijom.2022.03.001, PMID: 35288010

[49] Palmeri M Mehnert J Silk AW Jabbour SK Ganesan S Popli P . Real-world application of tumor mutational burden-high (TMB-high) and microsatellite instability (MSI) confirms their utility as immunotherapy biomarkers. ESMO Open. (2022) 7:100336. doi: 10.1016/j.esmoop.2021.100336, PMID: 34953399 PMC8717431

[50] Zheng M . Tumor mutation burden for predicting immune checkpoint blockade response: the more, the better. J ImmunoTherapy Cancer. (2022) 10:e003087. doi: 10.1136/jitc-2021-003087, PMID: 35101940 PMC8804687

[51] Pillay B Wootten AC Crowe H Corcoran N Tran B Bowden P . The impact of multidisciplinary team meetings on patient assessment, management and outcomes in oncology settings: A systematic review of the literature. Cancer Treat Rev. (2016) 42:56–72. doi: 10.1016/j.ctrv.2015.11.007, PMID: 26643552

[52] Taberna M Gil Moncayo F Jané-Salas E Antonio M Arribas L Vilajosana E . The multidisciplinary team (MDT) approach and quality of care. Front Oncol. (2020) 10:85. doi: 10.3389/fonc.2020.00085, PMID: 32266126 PMC7100151

[53] Roviello G Rodriquenz MG Aprile G D'Angelo A Roviello F Nobili S . Maintenance in gastric cancer: New life for an old issue? Crit Rev Oncology/Hematology. (2021) 160:103307. doi: 10.1016/j.critrevonc.2021.103307, PMID: 33753249

[54] Moehler M Högner A Wagner AD Obermannova R Alsina M Thuss-Patience P . Recent progress and current challenges of immunotherapy in advanced/metastatic esophagogastric adenocarcinoma. Eur J Cancer. (2022) 176:13–29. doi: 10.1016/j.ejca.2022.08.023, PMID: 36183651

